# Hybrid Membrane-Derived Nanoparticles for Isoliquiritin Enhanced Glioma Therapy

**DOI:** 10.3390/ph15091059

**Published:** 2022-08-26

**Authors:** Wenwan Shi, Xia Cao, Qi Liu, Qin Zhu, Kai Liu, Tianwen Deng, Qingtong Yu, Wenwen Deng, Jiangnan Yu, Qilong Wang, Ximing Xu

**Affiliations:** 1 Department of Pharmaceutics, School of Pharmacy, Centre for Nano Drug/Gene Delivery and Tissue Engineering, Jiangsu University, Zhenjiang 212013, China; 2 Medicinal Function Development of New Food Resources, Jiangsu Provincial Research Center, Zhenjiang 212013, China

**Keywords:** red blood cell membrane, nanoparticle, antitumor, mechanism

## Abstract

Due to the obstruction and heterogeneity of the blood-brain barrier, the clinical treatment of glioma has been extremely difficult. Isoliquiritigenin (ISL) exhibits antitumor effects, but its low solubility and bioavailability limit its application potential. Herein, we established a nanoscale hybrid membrane-derived system composed of erythrocytes and tumor cells. By encapsulating ISL in hybrid membrane nanoparticles, ISL is expected to be enhanced for the targeting and long-circulation in gliomas therapy. We fused erythrocytes with human glioma cells U251 and extracted the fusion membrane via hypotension, termed as hybrid membrane (HM). HM-camouflaged ISL nanoparticles (ISL@HM NPs) were prepared and featured with FT-IR, SEM, TEM, and DLS particle analysis. As the results concluded, the ISL active pharmaceutical ingredients (APIs) were successfully encapsulated with HM membranes, and the NPs loading efficiency was 38.9 ± 2.99% under maximum entrapment efficiency. By comparing the IC50 of free ISL and NPs, we verified that the solubility and antitumor effect of NPs was markedly enhanced. We also investigated the mechanism of the antitumor effect of ISL@HM NPs, which revealed a marked inhibition of tumor cell proliferation and promotion of senescence and apoptosis of tumor cells of the formulation. In addition, the FSC and WB results examined the effects of different concentrations of ISL@HM NPs on tumor cell disruption and apoptotic protein expression. Finally, it can be concluded that hybridized membrane-derived nanoparticles could prominently increase the solubility of insoluble materials (as ISL), and also enhance its targeting and antitumor effect.

## 1. Introduction

Isoliquiritin (ISL) is a natural chalcone extracted from licorice, belonging to the flavonoids. Flavonoids generally have antioxidant [1], free radical scavenging [2], anticancer [3], antiviral [4], anti-inflammatory [5], and other pharmacological effects [6], and are increasingly applied [7]. It has been shown that ISL exerts anti-inflammatory activity by inhibiting the production of prostaglandin E2 (PGE2) and interleukin-6 (IL-6) by macrophages [8]. Inflammation plays a major role in the development of malignant tumors. Flavonoids are considered to be good chemopreventive drug candidates with low toxicity and significant anti-tumor activity in the therapy of inflammation-associated tumors [9]. Recent reports had identified the anti-tumor activity of ISL. Zhang [10] et al. found that ISL inhibited cell proliferation, induced cell apoptosis, and caused G2/M (late DNA synthesis and late division) cell cycle arrest at 25–50 μM in vitro. They also found that ISL could significantly alter the signaling pathways of cell cycle, DNA damage, and apoptosis signaling pathways through microarray transcriptional profiling. Zhao [11] et al. demonstrated that ISL could prevent ethoxyethane (AOM)-induced colon cancer by inhibiting M2 macrophage polarization in animal models. The mechanisms of ISL in tumor cells include induction to apoptosis, inhibition of tumor cell proliferation, regulation of autophagy, and anti-angiogenesis [12]. However, the application of ISL in clinical is still limited due to its poor hydrophobicity and insolubility.

Drug delivery across the brain is difficult due to the blood-brain barrier (BBB), so most drugs, antibiotics, and neuropeptides cannot overcome the BBB. Nanoparticles have the potential to treat extremely aggressive brain tumors by endocytosis into endothelial cells of brain capillaries [13]. They can also effectively be accumulated in tumor sites through the enhanced permeability and retention (EPR) effect, promising to be delivery vehicles for anti-brain cancer drugs [14,15]. However, nanodrugs still face challenges, involving poor drug loading efficiency, rapid blood circulation clearance, low antitumor efficiency, and high recurrence risk [16]. More importantly, nanomedicine targeting glioma is hampered by the heterogeneity of glioma and the blood-brain barrier, which causes the non-selective distribution of drugs in the brain [17]. Therefore, designing an ideal targeting drug delivery system is not only necessary but also a long-term challenge in glioma therapy [18].

Recent studies have shown successful targeted therapy of brain tumors using nanocarriers, viruses, and cell-derived nanoparticles [19,20]. Among them, cellular delivery systems offer many advantages over artificial nanocarriers and microcarriers, such as immunogenicity and low cytotoxicity, targeting injured tissues, and prolonged internal circulation and half-life [21]. Currently, numerous types of membranes including cancer cells, bacteria, or macrophages have been applied in the modification of NPs for drug delivery [22,23]. Erythrocytes, monocytes, and neutrophils have long cycles and specific tropism to injured tissues, which can be also exploited for drug delivery therapy [24].

Red blood cells (RBCs), a kind of blood cell, have immune function and are the most important oxygen transport medium in vertebrates [25]. RBCs are a potential natural carrier system for targeted drugs due to their excellent biomechanical flexibility, non-immunogenicity, biosecurity, abundant sources, and immunosuppressive ability to escape phagocytosis by reticuloendothelial cells [26,27,28]. For example, due to the presence of “self-labeling” (e.g., Cluster of Differentiation 47 (CD47) protein), RBC membrane (RBCM) coating allows for a variety of nanoparticles to avoid immune reaction in vivo [29]. Since mature erythrocytes lack a nucleus and most organelles, RBCs can provide maximum space for oxygenated hemoglobin, by simple hypotonicity and centrifugation, termed as red blood cell ghosts or RBCM. During different hypotonic hemolysis procedures or through their coupling with the cell surface, it can effectively load a broad spectrum of drugs, enzymes, other types of biologically-active substances, and inorganic nanoparticles (NPs) into the RBCM [30]. In addition, RBCM has a large number of active groups that could bind specific polypeptides or antibodies to the erythrocyte surface, which is highly beneficial for achieving the targeted therapies of various cells and tissues. This property can be effectively deployed for crossing biological barriers including the BBB [31].

In nanodrug delivery systems, surface modification with bio-membranes allows the transfer of intact membranes and peripheral proteins to nanoparticles [16,32]. The bio-membranes coated nanoparticles exhibit properties specific to the source cell. Thus, by fusing membrane material from two different cells, combinatory functions could be achieved [33]. Dehaini and his coauthors successfully fused RBC membrane and platelet membrane, the reported method of bestowing nanoparticles with enhanced functionality provides a facile and natural alternative to synthetic post-functionalization strategies [33]. Based on the unique properties of red blood cell and tumor cell membranes, Qin [34] and his co-authors fused RBCM with MCF-7 cell membranes and fabricated an RBCM hybrid membrane-camouflaged melanin nanoparticle (Melanin@RBC-M) platform. The fused Melanin@RBC-M exhibited prolonged blood circulation and simultaneous homotypic targeting to source MCF-7 cells. Similarly, Dongdong Wang [35] and his coauthors fused RBC and melanoma cells (B16-F10 cells) to create RBC-B16F10 hybrid membrane-coated copper sulfide NPs that exhibited excellent blood circulation and tumor-targeting ability.

However, no existing work has addressed ISL enveloped in the biofilm as a drug delivery system. Therefore, we proposed that ISL could be encapsulated in the combination of RBC and U251 hybrid membrane nanoparticles, which in turn might promote anti-glioma efficacy through membrane fusion.

## 2. Results and Discussion

**Figure 1:**
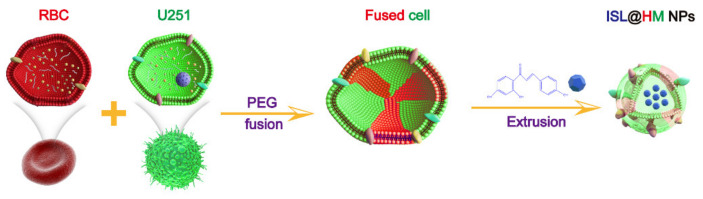
Illustration of the preparation of drug loaded ISL@HM NPs.

Nanotechnology has emerged as a promising solution for the elimination of cancer, but nanoparticles are rapidly cleared in vivo due to the lack of specificity for tumor cells [36]. Cell membrane coating is a powerful way to enhance the utility of nanoparticles. Nanoparticles coated with cell membrane could present a similar long circulation and target delivery as the original cells [24], especially for the red blood cells [37]. To improve the antitumor and brain targeting properties of ISL, here we report a facile and effective method for preparing HM by fusion of different types of cells. The ISL@HM NPs were characterized, and the anti-tumor effect was investigated. Figure 1 shows the preparation process of ISL@HM NPs.

### 2.1. Hybrid Membrane Preparation

**Figure 2:**
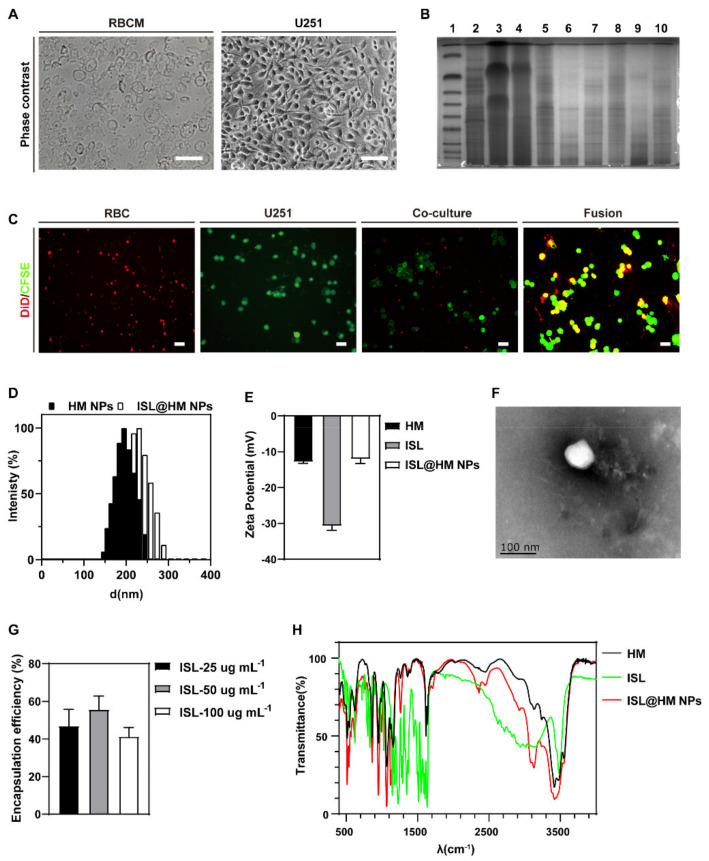
(**A**) cell morphology of RBC and adherent U251 cells. (**B**) SDS−PAGE protein analysis of Marker (1), U251 cells (2), RBCs (3), hybrid cells (4), U251 membrane (5), RBC membrane (6), hybrid membrane (7), U251 NPs (8), RBC NPs (9) and HM NPs (10). (**C**) Fluorescence images of RBC membrane, U251 membrane, a mixture of RBC membrane and U251 membrane, and the hybrid membrane. Red represents the fluorescence of DiD dye and green represented the fluorescence of CFSE dye (scale bars = 2 μm). (**D**) Size distributions of HM NPs and ISL@HM NPs in PBS. (**E**) Surface charge of HM vesicles, ISL API, and ISL@HM NPs. (**F**) TEM images of ISL@HM NPs with potassium phosphotungstate staining (scale bar = 100 nm). (**G**) ISL loading capacity of ISL@HM NPs when the ISL concentration was 25, 50, and 100 µg·mL^−1^, respectively. (**H**) FT-IR spectrum of HM, ISL API, and ISL@HM NPs.

The first step in the preparation of biomimetic nanoparticles was to prepare HM. There were two methods for preparing HM here: the first approach is to fuse cells before extracting the HM, and the second one is to fuse the two types of membranes after extracting cell membranes [38]. Cells fusion before HM preparation allows better retention of cell surface antigens [39]. Firstly, we isolated RBCs and U251 as shown in Figure 2A separately. The abundance of red blood cells in the body and the rapid proliferation of cancer cells make these two fused cells more easily accessible. The protein of RBC, U251, and hybrid membrane was subjected to the SDS-PAGE, respectively. As shown in Figure 2B, HM retains characteristic proteins inherited from RBC and U251 membranes. To further verify the successful fusion of RBC and U251, RBCs were stained with red 1,1′-Dioctadecyl-3,3,3′,3′-Tetramethylindodicarbocyanine,4-chlorobenzenesulfonate salt (DiD), and U251 were stained with green 5(6)-Carboxyfluorescein diacetate succinimidyl ester (CFSE). As shown in Figure 2C, significant colocalization between fluorescence signals derived from HM, while negligible colocalization could be found in physical coculture of RBC and U251 membrane. These results indicated the successful fusion of natural cell membranes from two different origins. Compared to erythrocyte/cancer single cell membrane, the hybrid cell membrane expressing CD47 membrane protein and self-recognition molecules, from erythrocytes and cancer cells, provides remarkable features to the synthetic vehicles, such as immune evasion, long-term circulation, and homotypic targeting [40]. In this paper, polyethylene glycol (PEG) 2000 was used to induce cell fusion, which PEGylation of the membrane during this process has the potential to further increase the circulating half-life. This is possibly due to PEG interference with protein adsorption on membrane coating defects [41]. Therefore, NPs prepared by fusion of RBC and U251 membrane are expected to deliver drugs to the brain and target the highly aggressive brain tumor U251.

### 2.2. Synthesis and Characterization of ISL@HM NPs

Drug encapsulation and surface coupling are two methods of HM drug delivery [42]. We designed the ISL@HM NPs by encapsulating insoluble ISL into HM with repeated squeezing using a microextruder. The average hydrodynamic diameter of HM NPs increased from 225.16 ± 12.68 nm to 264.59 ± 44.08 nm after loading of ISL (Figure 2D). Transmission electron microscopy (TEM) images can identify obvious layer coating structure in the ISL@HM NPs, clearly indicating the successful formation of ISL@HM NPs (Figure 2F). More TEM images of hybrid nanoparticles can be seen in Supplementary Materials. The zeta potentials of HM, ISL, and ISL@HM NPs were −13.33 ± 1.04 mV, −31.01 ± 1.26 mV, and −12.05 ± 1.23 mV, respectively (Figure 2E), and the highly negative core can be shielded by the less negative outer membrane surface [23,43]. It can be seen that the zeta potential of ISL@HM NPs rose to the same level as HM membrane, proving the successful encapsulation of the ISL. The bicinchoninic acid (BCA) protein kit showed the concentration of extracted HM membrane proteins was 242.76 ± 11.55 μg mL^−1^. The changes of chemical structure during the preparation of ISL@HM NPs were analyzed by Fourier infrared spectrometer (FT-IR). The FT-IR spectrum of ISL@HM NPs (Figure 2H) revealed the characteristic peaks of ISL were located at 3200–3000 cm^−1^ and 1250–500 cm^−1^ due to the C-H stretching vibration and fingerprint area on benzene [44]. At 1700 cm^−1^, the characteristic peak was enhanced by tensile vibration of the C=O bonds of ISL and HM [45]. The characteristic peaks of ISL at 3500–3300 cm^−1^ overlapped with the peaks of ISL@HM NPs, resulting in the peak enhancement of stretching vibration of the -OH groups [19]. The FT-IR spectrum of ISL@HM NPs displayed both characteristic peaks at the same time, indicating the successful encapsulation of ISL in HM NPs.

### 2.3. In Vitro Analyses

Poor solubility of isoliquiritin hinders it is in vivo application, while nano-preparation may help to improve this issue [46]. The drug loading ability of ISL@HM NPs in the presence of different concentrations of ISL was examined. As shown in Figure 2G, the drug encapsulation rates of ISL@HM NPs were 46.95%, 55.63%, and 41.32%, respectively, in the presence of 25 μg mL^−1^, 50 μg mL^−1^, and 100 μg mL^−1^ ISL. The results demonstrated that the HM had good encapsulation rates at 50 μg mL^−1^ ISL, so 50 μg mL^−1^ of ISL was selected for subsequent experiments, while NP’s loading capacity was 38.9 ± 2.99% under these conditions.

**Figure 3:**
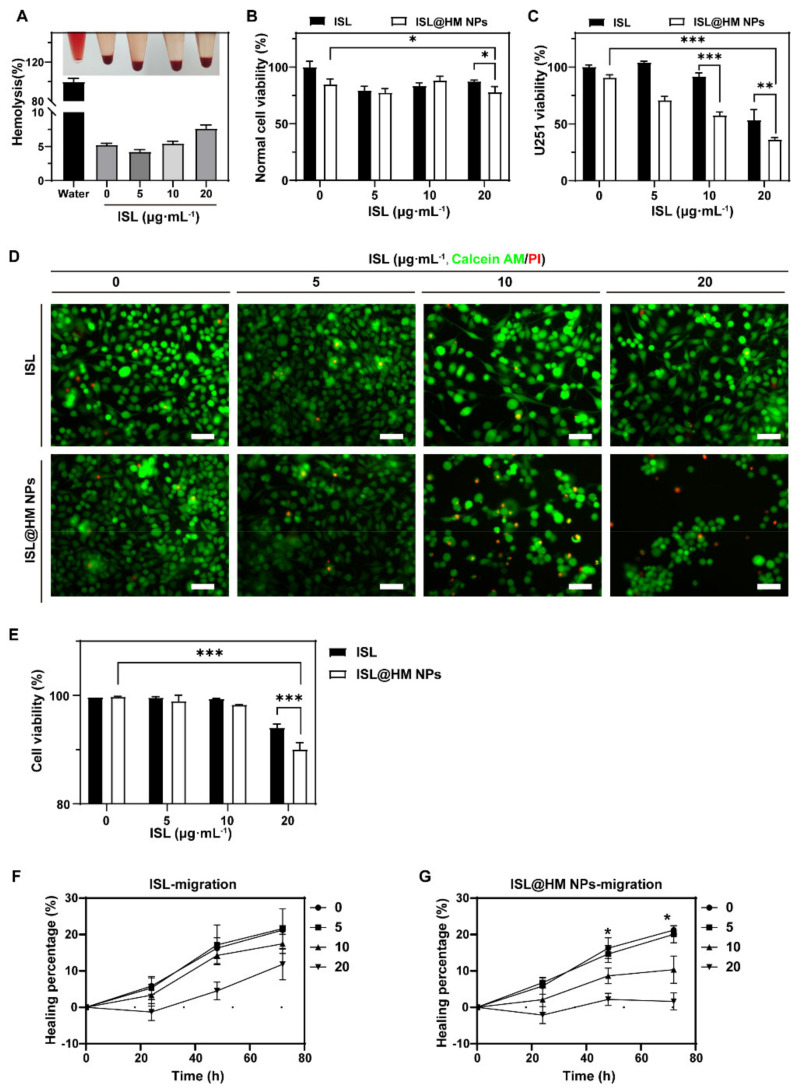
(**A**) Hemolysis quantification of HM at various concentrations of ISL@HM NPs. Cytotoxicity of ISL@HM NPs to HFF cells (**B**) and U251 cells (**C**,**D**) Live/dead staining and (**E**) the cell livability of U251 cells after different treatments (scale bar = 50 μm). The cell migration rate of U251 cells after different concentrations of ISL (**F**) and ISL@HM NPs (**G**) treatments. (*n* = 3; mean ± s.d. *** *p* < 0.001, ** *p* < 0.01 and * *p* < 0.05, as determined by unpaired, two−tailed Student’s *t*-tests. *n* refers to biological replicates.)

The hemolysis assay was performed to explore the hemocompatibility of the NPs. The hemolysis rate of ISL@HM NPs was lower than 8% after 6 h incubation with different concentrations of ISL (Figure 3A), which is similar to the research in other labs [19,47], indicating that ISL@HM NPs have high biosafety and biocompatibility, which is conducive for the practical application.

### 2.4. Cellar Examination

We also investigated the cytotoxicity of different NPs with different cells. 3-(4,5-Dimethylthiazol-2-yl)-2,5-diphenyltetrazolium bromide (MTT) assay results revealed that cell viability was more than 78% after treatment with different concentrations of ISL@HM NPs (0, 5, 10, 20 μg·mL^−1^) in HFF cells (Figure 3B), which indicated that ISL@HM NPs had high biosafety and biocompatibility in transformation by lentiviral transduction cells. Meanwhile, after 72 h incubation with U251 cells, cell viability was reduced to 38% at the concentration of 20 μg mL^−1^ (Figure 3C). This suggested ISL@HM NPs had good antitumor effect, which may be due to HM enabling nanoparticles with excellent homologous targeting.

With the advanced research of flavonoids and other traditional Chinese medicines, isoliquiritin has shown the ability to anti-tumor [48,49] and induction of tumor cells to differentiate into normal cells [50]. Subsequently, we further investigated the antitumor ability of ISL@HM NPs in vitro. We observed and calculated the live and dead viability with Calcein AM/propidium iodide (C-AM/PI) dual fluorescent dyes stained U251. With the increase of ISL, the green fluorescence was significantly reduced, and red fluorescence increased in ISL@HM NPs group compared to free ISL (Figure 3D,E). These results suggested a significant antitumor effect of ISL@HM NPs in vitro.

Wound healing is a simple method to study cell migration or tumor invasion. In Figure 3F,G, the damage-repair curves of U251 cells at different concentrations of ISL in ISL@HM NPs were continuously detected for 72 h. The results showed that the healing percentages of U251 cells in ISL@HM NPs group were 6%, 45.2%, and 75%, respectively, which were significantly higher than the blank group (2.5%). Our study demonstrated that ISL and ISL@HM NPs significantly restrain U251 cell migration, suggesting an inhibitory role of ISL@HM NPs in the treatment of brain tumors.

### 2.5. Primarily Anti-Tumor Mechanism Analysis

**Figure 4:**
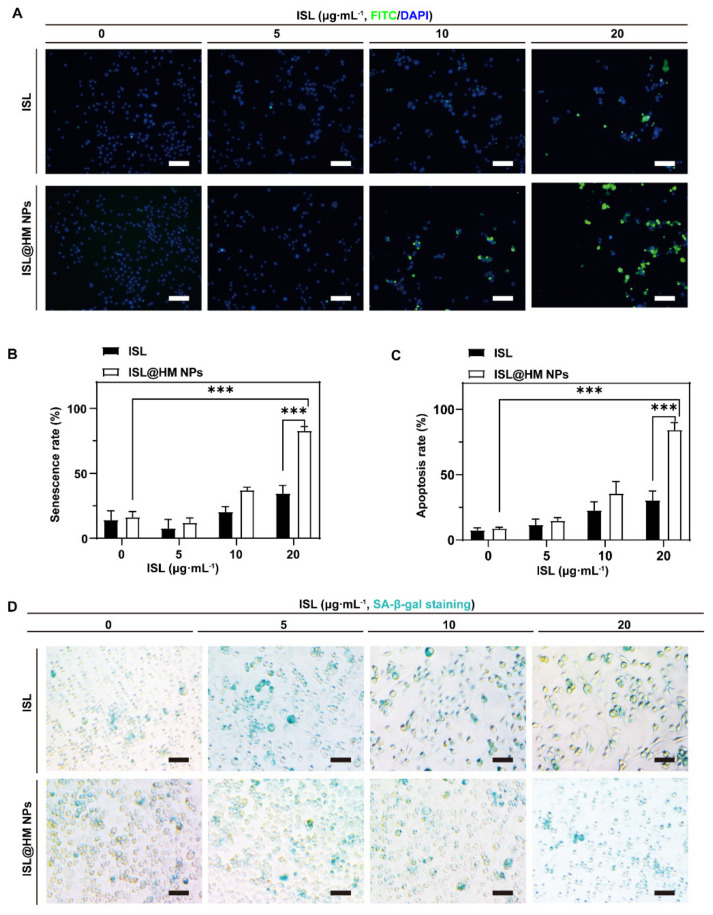
Fluorescence images (**A**) and quantitative assay (**B**) of TUNEL staining after different treatments with U251 cells (scale bar = 50 μm). Fluorescence images (**D**) and quantitative assay (**C**) of β−Galactosidase staining after different treatments with U251 cells (scale bar = 50 μm). (*n* = 3; mean ± s.d. *** *p* < 0.001, as determined by unpaired, two−tailed Student’s *t*-tests. *n* refers to biological replicates.)

Cells responding to DNA damage implement complex adaptive programs that often culminate in one of two distinct outcomes: apoptosis or senescence [51]. Here, we examined cell apoptosis by the green fluorescence change of intracellular medium using the terminal deoxynucleotidyl transferase (TdT)-mediated dUTP nick-end labeling (TUNEL) assay kit to detect. On the other hand, we employed a β-galactosidase staining kit to detect the changes of SA-β-Gal (senescence-associated β-galactosidase) activity level during cell senescence induced by ISL@HM NPs. A characteristic feature of apoptosis is DNA fragmentation. This fragmentation can be detected by TUNEL of DNA in dying cells [52,53]. As depicted in Figure 4A, the green fluorescence in ISL@HM NPs treated cells was greatly increased, while Figure 4B exhibited a rate of apoptosis cells of 84.41% in the 20 μg mL^−1^ ISL@HM NPs treated group, which was significantly higher than the apoptosis rate of 7.79% in blank group. These results suggested that ISL@HM NPs have considerable apoptotic ability of tumor cells in vitro through DNA damage, which is consistent with previous work [54]. Cellular senescence has been identified as one of the mechanisms mediating the anticancer effects of chemotherapies [55]. As illustrated in Figure 4C,D, the senescence rate of 82.93% of cells in 20 μg mL^−1^ ISL@HM NPs treated group, which was dramatically higher than the early senescence rate of 14.31% in the blank group. These results suggested that cell senescence may contribute to the anti-tumor and growth-inhibiting effects of ISL@HM NPs.

**Figure 5:**
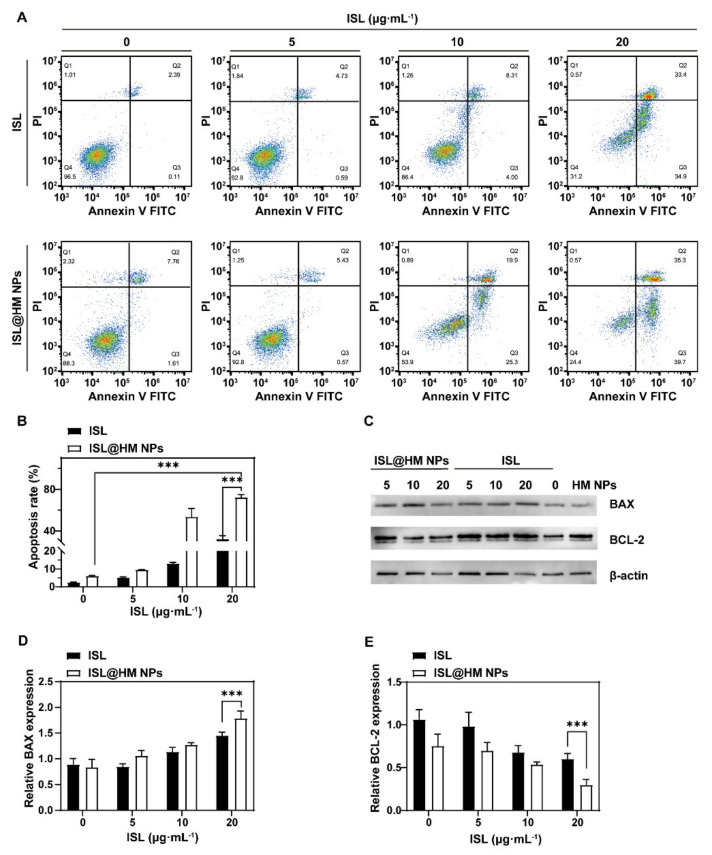
Annexin V−FITC/PI apoptosis assay (**A**) and quantitative analysis (**B**) of U251 cells after different treatments. Western blotting analysis (**C**) and quantitative analysis (**D**,**E**) of Bcl−2 and BAX expression in U251 cells after different treatments. (*n* = 3; mean ± s.d. *** *p* < 0.001, as determined by unpaired, two−tailed Student’s *t*-tests. *n* refers to biological replicates.)

The mechanism of ISL@HM NPs promoting apoptosis of U251 cells was further investigated with a fluorescence-activated cell sorter (FACS). U251 cells were stained with Annexin V-FITC and propidium iodide (PI) to distinguish and quantify non-apoptotic cells (Annexin V-FITC negative/PI negative), early apoptotic cells (Annexin V-FITC positive/PI negative), late apoptotic/necrotic cells (Annexin V-FITC positive/PI positive) and dead cells (Annexin V-FITC negative/PI positive). The findings of Annexin V-FITC/PI apoptosis assay disclosed that the total number of early apoptotic and late apoptotic U251 cells at different concentrations of the ISL@HM NPs treated group was 9.52% ± 0.14, 53.6% ± 8.03, 72.3% ± 2.61, respectively, which were distinctly superior to the blank group with an apoptotic rate of 2.57% ± 0.07 (Figure 5A,B). We found that the apoptotic phenotype occurred in more cells than the necrotic phenotype, therefore, apoptosis may be the prevalent response to ISL treatment in U251 cells [56]. These results clearly implied that ISL@HM NPs can trigger apoptotic cell death, which can also be potent inducers of senescence.

Finally, we observed the apoptosis-promoting ability of ISL@HM NPs at molecular level. B cell lymphoma 2 (Bcl-2) and Bcl-2 associated X (Bax) protein belong to the Bcl-2 family, where Bcl-2 is the anti-apoptotic protein and Bax is the pro-apoptotic protein. The balance of these two proteins determines whether a cell undergoes apoptosis [57,58]. We analyzed the location and depth of staining of the Western blot protein bands which reflect the expression of Bcl and BAX in treated U251. Bcl-2 protein level was suppressed significantly and Bax protein level was increased dramatically in the ISL@HM NPs group compared to the control (Figure 5C–E). The changes in the expression in these proteins were consistent with the trend of cell apoptosis, proving that ISL@HM NPs induced apoptosis. Previous studies have demonstrated that ISL-induced cell apoptosis is associated with disruption of mitochondrial function [49]. A cluster of proteins belonging to the Bcl-2 family localizes to the outer membrane of mitochondria to regulate cell apoptosis [57]. Therefore, ISL@HM NPs may induce U251 apoptosis via mitochondrial signaling [59].

## 3. Materials and Methods

### 3.1. Materials

Isoliquiritigenin (ISL) and 3-(4,5-Dimethylthiazol-2-yl)-2,5-diphenyltetrazolium bromide (MTT) were purchased from Shanghai Aladdin Biochemical Technology Co., Ltd. (Shanghai, China). U251 cells were obtained from the National Collection of authenticated cell cultures (Shanghai, China). Human foreskin fibroblasts (HFF) were obtained from Zhong Qiao Xin Zhou Biotechnology Co., Ltd. (Shanghai, China). Fetal bovine serum (FBS) was purchased from Thermo Fisher Scientific Co., Ltd. (Shanghai, China). DF12 1:1 and DMEM media were purchased from Cytiva life sciences Co., Ltd. (Beijing, China). Chemical reagents, including TMA-DPH, Calcein-AM, 5(6)-carboxyfluorescein diacetate succinimidyl ester (CFSE), propidium iodide (PI) were purchased from MedChemExpress Biotechnology Co., Ltd. (Shanghai, China). RIPA lysis buffer, phenylmethylsulfonyl fluoride (PMSF), and β-galactosidase staining kit were purchased from Solarbio Science & Technology Co., Ltd. (Beijing, China). Bicinchoninic Acid (BCA) protein assay kits were purchased from Novazin Biotechnology Co., Ltd. (Nanjing, China). Terminal deoxynucleotidyl transferase mediated 2′-deoxyuridine 5′-triphosphate nick-end labeling (TUNEL) test kit, 1,1′-Dioctadecyl-3,3,3′,3′-Tetramethylindodicarbocyanine,4-chlorobenzenesulfonate salt (DID) and pepstatin A were purchased from Beyotime Biotechnology Co., Ltd. (Shanghai, China). Annexin Ⅴ-FITC/PI double staining apoptosis detection kits were purchased from Nanjing Jiancheng Bioengineering Institute Co., Ltd. (Nanjing, China). NaCl, Na_2_HPO_4_, NaH_2_PO_4_, and ethylene diamine tetraacetic acid (EDTA) were purchased from Sinopharm Chemical Reagent Co., Ltd. (Shanghai, China). PAGE Gel Fast Preparation Kits and Coomassie Blue Fast Staining were purchased from Epizyme Biotech Co., Ltd. (Shanghai, China). B cell lymphoma 2 (Bcl-2) and Bcl-2 associated X (Bax) antibodies were purchased from Abcam Trading Co., Ltd. (Shanghai, China). SD rats were obtained from Jiangsu university laboratory animal research center (Zhenjiang, China). All other reagents were of analytical purity and used without further purification.

### 3.2. Preparation of Hybrid Membrane

The animal studies were approved by the Institutional Animal Care and Use Committee of Jiangsu University (Jiangsu, China; protocol code UJS-IACUC-2021042002 and approved on 31 July 2022). Firstly, whole blood collected from SD male rats was centrifuged at 1400× *g* for 3 min, and RBCs were separated. The isolated RBC was washed with ice-cold phosphate buffer saline (PBS) solution until the supernatant became colorless to obtain RBCM. RBCM were mixed with U251 cells in a 10:1 ratio, and the mixed cells were washed three times with serum-free Dulbecco’s modified eagle medium (DMEM) medium and centrifuged at 1000× *g* for 5 min. The bottom of the tube was flicked to mix the two cells evenly. Fusion was induced by dropwise addition of pre-warmed 50% PEG 2000 at 37 °C. PEG fusion was terminated by adding pre-warmed DMEM medium containing 15% serum to the tube after one minute. After being centrifuged at 1000× *g* for 5 min, RBC and U251 fused cells were obtained.

Next, the HM was prepared by hypotonic dissolution. Briefly, the hybrid cells were incubated in ultrapure water at 4 °C to induce osmotic lysis and liberation of the cell nuclei (after about 20 min, monitored by microscope). HM was separated from unbroken cells and nuclei by centrifugation at 2000× *g* for 20 min. The obtained was concentrated by ultrafiltration tube (100 kDa) through centrifuged at 4000× *g* for 20 min. All procedures were performed on ice. The concentrated HM was diluted in PBS and stored at −80 °C. BCA protein assay kit was performed to quantify the concentration of HM proteins.

### 3.3. Synthesis of ISL@HM NPs

The hybrid membrane camouflaged isoliquiritin nanoparticles (ISL@HM NPs) were prepared by extrusion method [29,36]. Briefly, 0.9 mL of PBS buffer or medium was added to 100 μL of HM solution (0.25 m44g mL^−1^). After that, 5 μL of ISL solution was added for final concentration of 10 mg mL^−1^. The mixture was sonicated for 20 min at room temperature (RT). ISL@HM NPs were formed using a micro-syringe (Avanti, Birmingham, AL, USA) extruded 15 times by squeezing through 400 nm polycarbonate porous membranes.

### 3.4. Characterization of Hybrid Membrane

Sodium dodecyl sulfate-polyacrylamide gel electrophoresis (SDS-PAGE) was used to characterize the membrane protein. Briefly, RBC membrane, U251 membrane, and HM were lysed with radio immunoprecipitation assay (RIPA) lysis buffer and total protein concentration was determined by BCA protein assay kit. All samples with equivalent protein amounts were loaded on 12% SDS-PAGE gel and electrophoresed. Then the resultant gel was stained in Coomassie Blue staining for 2 h and washed overnight for subsequent imaging with luminescence imaging system (CLINX, CHN).

Additionally, the fused cell membrane was labeled and observed under a fluorescence microscope. Briefly, U251 and RBCs were stained with CFSE and DID, separately. The two labeled cells were fused as described above. CFSE was excited by a 488 nm laser and the green emission was collected at 518 nm. DiD was excited by a 644 nm laser and the red emission was collected at 665 nm.

### 3.5. Characterization of ISL@HM NPs

ISL@HM NPs were characterized by TEM (Zeiss, Jena, Germany), dynamic light scattering (DLS, Brookhaven, GA, USA), and FT-IR (Nicolet, Madison, WI, USA).

### 3.6. In Vivo Exploration

#### 3.6.1. Loading Efficiency (LE) and Encapsulation Efficiency (EE)

ISL@HM NPs were demulsified with ethanol and centrifuged at 400× *g* for 5 min using an ultrafiltration tube (10,000 kDa) to remove unencapsulated ISL from the supernatant. To quantify the *LE* and *EE* of ISL, different concentrations of ISL (25, 50, 100 μg mL^−1^) were loaded into ISL@HM NPs following a similar procedure. The supernatant was then demulsified with ethanol and measured by a microplate reader (BioTek, Winooski, VT, USA). The membrane protein content in ISL@HM NPs was measured using a BCA protein assay kit. The *LE* and *EE* results of ISL were calculated according to Equations (1) and (2).

(1) $$LE\left(\%\right)=\frac{M_{E}}{M_{E}+M_{P}}\times 100\%$$

(2) $$EE\left(\%\right)=\frac{M_{E}}{M_{N}}\times 100\%$$

where *M_E_* is the mass of encapsulated ISL, *M_N_* is the mass of ISL, and *M_P_* is the mass of membrane protein in ISL@HM NPs.

#### 3.6.2. Hemolysis Assay

Mouse whole blood was centrifuged at 400× *g* for 5 min and washed five times with PBS solution to extract pure erythrocytes. Afterward, 500 μL of 4% erythrocytes (*v*/*v*) was mixed with 500 μL of ISL@HM NPs solution at various concentrations (5, 10, and 20 mg mL^−1^) and incubated at 37 °C for 8 h. The erythrocytes were mixed with pure water and 100% hemolyzed. The absorbance of the samples supernatants at 540 nm was measured on a microplate reader. The percentage of hemolysis was calculated according to Equation (3).

(3) $$\mathrm{Hemolysis}\;\left(\%\right)=\frac{I}{I_{0}}\times 100\%$$

where *I* represent the absorbance of supernatant for erythrocytes with ISL@HM NPs, and *I*_0_ is the absorbance of erythrocytes after complete hemolysis in pure water.

### 3.7. Cellular Assay

#### 3.7.1. Cell Culture

U251 and HFF cells were cultured in DMEM medium containing 10% Fetal Bovine Serum (FBS) and 1% streptomycin/penicillin (PS). Cells were cultured at 37 °C in an incubator with 5% CO_2_.

#### 3.7.2. Cell Viability

The cytotoxicity of ISL@HM NPs against U251 cells and HFF cells was determined by MTT assay. U251 was seeded in a 96-well plate at a density of 1 × 10^4^ cells per well and incubated for 24 h. The original medium was refreshed with serum free medium containing different concentrations of ISL (0, 5, 10, 20 μg·mL^−1^) in free ISL and ISL@HM NPs, respectively. Meanwhile, untreated cells were detected as a control. After cells were treated for 72 h, 20 μL MTT (5 mg mL^−1^) solution was added to each well. Cells were incubated for another 4 h. The supernatants of each well were discarded and 100 μL dimethyl sulphoxide (DMSO) was added. The absorbance at 490 nm was measured using a microplate reader. Cell viability was calculated according to Equation (4).

(4) $$\mathrm{Cell}\;\mathrm{viability}=\frac{A1-Ac}{A0-Ac}\times 100\%$$

where *A*1 is the absorbance value of the cells treated with different concentrations of ISL@RBC, *A*0 is the absorbance value of cells without treatment, and *Ac* is the absorbance value of the MTT medium solution at 490 nm.

#### 3.7.3. Live/Dead Assay

The antitumor efficacy was further explored by live/dead staining. U251 cells were seeded in a 96-well plate at a density of 1 × 10^4^ cells per well and incubated at 37 °C in 5% CO_2_ for 24 h. The original medium was refreshed with serum free medium containing different concentrations of ISL (0, 5, 10, 20 μg·mL^−1^) in free ISL and ISL@HM NPs. The medium of each well was discarded after 72 h of incubation, and 100 μL fresh Serum free medium with 0.2 μL of Calcein-AM (10 μM) solution and 0.5 μL PI staining solution (15 μM) was added into each well, stained for 10 min. All the cells were washed with PBS in triplicated and imaged using inversed fluorescent microscope.

#### 3.7.4. Wound Healing Assay

U251 cells were seeded in a 48-well plate at a density of 2 × 10^4^ cells per well and incubated for 24 h at 37 °C in 5% CO_2_. The cell culture plate was scratched vertically with a 20 μL sterilized pipette tip and washed with PBS in triplication to remove the floating cells, and the slits were clearly visible. The serum free medium containing different concentrations of ISL (0, 5, 10, 20 μg mL^−1^) in free ISL and ISL@HM NPs was replaced in each well, separately. Cells were cultivated in a 5% CO_2_ incubator at 37 °C. Cells scratches were observed and recorded using a microscope at different time points (24, 48, 72 h).

### 3.8. Related Mechanism Analysis

#### 3.8.1. TUNEL Test Kit Apoptosis Assay

U251 cells were seeded at a density of 1 × 10^4^ in a 96-well plate and cultivated for 24 h. Next, different concentrations of ISL (0, 5, 10, 20 μg mL^−1^) in free ISL and ISL@HM NPs added to fresh serum free medium and incubated for another 72 h. For the apoptosis assay, the cells were stained with TUNEL test kit. Finally, the treated cells were imaged under inverted fluorescent microscope.

#### 3.8.2. β-Galactosidase Staining Kit

U251 cells were seeded at a density of 2 × 10^4^ cells per well in a 48-well plate and cultivated for 24 h. Next, different concentrations (0, 5, 10, 20 μg mL^−1^) of ISL in free ISL and ISL@HM NPs were mixed with fresh serum free medium, added to each well, and cultivated for another 72 h. For the senescence assay, cells were stained with a β-galactosidase staining kit. Finally, the treated cells were imaged under inverted fluorescent microscope. All the experiments were replicated in triplicate.

#### 3.8.3. Apoptosis Assay

U251 cells were seeded at a density of 1 × 10^5^ cells per well in a 6-well plate and cultivated for 24 h. Next, different concentrations (0, 5, 10, 20 μg mL^−1^) of ISL@HM NPs were added to fresh serum-free medium and incubated for another 72 h. For the apoptosis assay, the cells were stained with Annexin V-FITC/PI solution. Finally, the treated cells were detected by flow cytometry (BD, Franklin Lakes, NJ, USA) and analyzed via flowjo (v10.8.1).

#### 3.8.4. Western Blotting

U251 cells were inoculated in a 6-well plate at a density of 1 × 10^5^ per well and cultivated for 24 h, followed by the addition of different concentrations of ISL (0, 5, 10, 20 μg mL^−1^) in ISL@HM NPs in fresh serum free medium. After further incubation for another 72 h, the cells were harvested and lysed with RIPA lysis buffer on ice. Finally, western blotting was performed to detect the expression level of Bcl-2 and Bax in U251 cells. The results were detected by a Chemiscope 3600 mini luminescence imaging system (CLINX, CHN).

### 3.9. Statistical Analysis

All the samples were conducted at least three parallel trials. Graphical and statistical analyses were performed using GraphPad Prism 8 and ImageJ software. Differences were considered significant when *p* < 0.05.

## 4. Conclusions

In summary, we presented a theoretical and experimental study of the ISL@HM NPs. Firstly, we successfully prepared HM and synthesized ISL@HM NPs, which were shown good biocompatibility in hemolysis experiments. The results of the MTT assays and C-AM/PI staining indicated that ISL@HM NPs could inhibit the proliferation of U251 in a dose-dependent manner. Furthermore, the results of the wound healing illustrated that ISL@HM NPs suppressed the migration of U251 cells in vitro. We found ISL@HM NPs distinctly elevated the percentage of apoptosis and senescence in U251 cells shown by TUNEL assays, β-galactosidase staining, and flow cytometry analysis. Furthermore, the expression of apoptosis-associated proteins was measured to understand the molecule mechanisms. The levels of Bax were significantly elevated, while Bcl-2 was remarkably reduced compared with the control group. These results suggest that ISL@HM NPs can promote U251 cell apoptosis and senescence. However, more in vivo anti-tumor experiments should be studied to verify the in vitro findings. Further research on the role and mechanism of nanoparticles in vivo will be done in our further studies. The combination of hybrid membranes, such as RBC and tumor cells, could improve the controllability and targeting of drug delivery systems

## Data Availability

Data is contained within the article and Supplementary Materials.

## Supplementary Materials

The following supporting information can be downloaded at: https://www.mdpi.com/article/10.3390/ph15091059/s1.

## References

[1] Salachna P., Pietrak A., Lopusiewicz L. (2021). Antioxidant Potential of Flower Extracts from *Centaurea* spp. Depends on Their Content of Phenolics, Flavonoids and Free Amino Acids. Molecules.

[2] Amić D., Stepanić V., Lučić B., Marković Z., Dimitrić Marković J. (2013). PM6 study of free radical scavenging mechanisms of flavonoids: Why does O-H bond dissociation enthalpy effectively represent free radical scavenging activity?. J. Mol. Model..

[3] Zhang Z., Yang L., Hou J., Tian S., Liu Y. (2021). Molecular mechanisms underlying the anticancer activities of licorice flavonoids. J. Ethnopharmacol..

[4] Badshah S., Faisal S., Muhammad A., Poulson B., Emwas A., Jaremko M. (2021). Antiviral activities of flavonoids. Biomed. Pharmacother..

[5] Al-Khayri J., Sahana G., Nagella P., Joseph B., Alessa F., Al-Mssallem M. (2022). Flavonoids as Potential Anti-Inflammatory Molecules: A Review. Molecules.

[6] Numakawa T., Odaka H. (2021). Brain-Derived Neurotrophic Factor Signaling in the Pathophysiology of Alzheimer’s Disease: Beneficial Effects of Flavonoids for Neuroprotection. Int. J. Mol. Sci..

[7] Wang J.R., Luo Y.H., Piao X.J., Zhang Y., Feng Y.C., Li J.Q., Xu W.T., Zhang Y., Zhang T., Wang S.N. (2019). Mechanisms underlying isoliquiritigenin-induced apoptosis and cell cycle arrest via ROS-mediated MAPK/STAT3/NF-kappaB pathways in human hepatocellular carcinoma cells. Drug Dev. Res..

[8] Kim J., Park S., Yun K., Cho Y., Park H., Lee K. (2008). Isoliquiritigenin isolated from the roots of Glycyrrhiza uralensis inhibits LPS-induced iNOS and COX-2 expression via the attenuation of NF-kappaB in RAW 264.7 macrophages. Eur. J. Pharmacol..

[9] Chahar M., Sharma N., Dobhal M., Joshi Y. (2011). Flavonoids: A versatile source of anticancer drugs. Pharmacogn. Rev..

[10] Zhang B., Lai Y., Li Y., Shu N., Wang Z., Wang Y., Li Y., Chen Z. (2018). Antineoplastic activity of isoliquiritigenin, a chalcone compound, in androgen-independent human prostate cancer cells linked to G2/M cell cycle arrest and cell apoptosis. Eur. J. Pharmacol..

[11] Zhao H., Zhang X., Chen X., Li Y., Ke Z., Tang T., Chai H., Guo A.M., Chen H., Yang J. (2014). Isoliquiritigenin, a flavonoid from licorice, blocks M2 macrophage polarization in colitis-associated tumorigenesis through downregulating PGE2 and IL-6. Toxicol. Appl. Pharm..

[12] Zhang J., Li X., Huang L. (2020). Anticancer activities of phytoconstituents and their liposomal targeting strategies against tumor cells and the microenvironment. Adv. Drug Deliv. Rev..

[13] Kreuter J. (2001). Nanoparticulate systems for brain delivery of drugs. Adv. Drug Deliv. Rev..

[14] Farokhzad O., Langer R. (2009). Impact of nanotechnology on drug delivery. ACS Nano.

[15] Hu C., Lei T., Wang Y., Cao J., Yang X., Qin L., Liu R., Zhou Y., Tong F., Umeshappa C.S. (2020). Phagocyte-membrane-coated and laser-responsive nanoparticles control primary and metastatic cancer by inducing anti-tumor immunity. Biomaterials.

[16] Chen W., Zeng K., Liu H., Ouyang J., Wang L., Liu Y., Wang H., Deng L., Liu Y.-N. (2017). Cell Membrane Camouflaged Hollow Prussian Blue Nanoparticles for Synergistic Photothermal-/Chemotherapy of Cancer. Adv. Funct. Mater..

[17] Gao H. (2017). Perspectives on Dual Targeting Delivery Systems for Brain Tumors. J. Neuroimmune Pharm..

[18] Wang C., Sun X., Cheng L., Yin S., Yang G., Li Y., Liu Z. (2014). Multifunctional theranostic red blood cells for magnetic-field-enhanced in vivo combination therapy of cancer. Adv. Mater..

[19] Luo L., Zeng F., Xie J., Fan J., Xiao S., Wang Z., Xie H., Liu B. (2020). A RBC membrane-camouflaged biomimetic nanoplatform for enhanced chemo-photothermal therapy of cervical cancer. J. Mater. Chem. B.

[20] Van’t Root M., Lowik C., Mezzanotte L. (2017). Targeting Nanomedicine to Brain Tumors: Latest Progress and Achievements. Curr. Pharm. Des..

[21] Su Y., Xie Z., Kim G.B., Dong C., Yang J. (2015). Design strategies and applications of circulating cell-mediated drug delivery systems. ACS Biomater. Sci. Eng..

[22] Zhang Q., Dehaini D., Zhang Y., Zhou J., Chen X., Zhang L., Fang R.H., Gao W., Zhang L. (2018). Neutrophil membrane-coated nanoparticles inhibit synovial inflammation and alleviate joint damage in inflammatory arthritis. Nat. Nanotechnol..

[23] Hu C.-M.J., Fang R.H., Wang K.-C., Luk B.T., Thamphiwatana S., Dehaini D., Nguyen P., Angsantikul P., Wen C.H., Kroll A.V. (2015). Nanoparticle biointerfacing by platelet membrane cloaking. Nature.

[24] Anselmo A.C., Mitragotri S. (2014). Cell-mediated delivery of nanoparticles: Taking advantage of circulatory cells to target nanoparticles. J. Control. Release.

[25] Cines D.B., Lebedeva T., Nagaswami C., Hayes V., Massefski W., Litvinov R.I., Rauova L., Lowery T.J., Weisel J.W. (2014). Clot contraction: Compression of erythrocytes into tightly packed polyhedra and redistribution of platelets and fibrin. Blood.

[26] Timin A., Litvak M., Gorin D., Atochina-Vasserman E., Atochin D., Sukhorukov G. (2018). Cell-Based Drug Delivery and Use of Nano-and Microcarriers for Cell Functionalization. Adv. Healthc. Mater..

[27] Gao L., Wang H., Nan L., Peng T., Sun L., Zhou J., Xiao Y., Wang J., Sun J., Lu W. (2017). Erythrocyte Membrane-Wrapped pH Sensitive Polymeric Nanoparticles for Non-Small Cell Lung Cancer Therapy. Bioconjug. Chem..

[28] Gao M., Liang C., Song X., Chen Q., Jin Q., Wang C., Liu Z. (2017). Erythrocyte-Membrane-Enveloped Perfluorocarbon as Nanoscale Artificial Red Blood Cells to Relieve Tumor Hypoxia and Enhance Cancer Radiotherapy. Adv. Mater..

[29] Zheng D., Yu P., Wei Z., Zhong C., Wu M., Liu X. (2020). RBC Membrane Camouflaged Semiconducting Polymer Nanoparticles for Near-Infrared Photoacoustic Imaging and Photothermal Therapy. Nano-Micro Lett..

[30] Kolesnikova T., Skirtach A., Möhwald H. (2013). Red blood cells and polyelectrolyte multilayer capsules: Natural carriers versus polymer-based drug delivery vehicles. Expert Opin. Drug Deliv..

[31] Allen T. (2002). Ligand-targeted therapeutics in anticancer therapy. Nat. Rev. Cancer.

[32] Wang Y., Zhang K., Qin X., Li T., Qiu J., Yin T., Huang J., McGinty S., Pontrelli G., Ren J. (2019). Biomimetic Nanotherapies: Red Blood Cell Based Core-Shell Structured Nanocomplexes for Atherosclerosis Management. Adv. Sci..

[33] Dehaini D., Wei X., Fang R.H., Masson S., Angsantikul P., Luk B.T., Zhang Y., Ying M., Jiang Y., Kroll A.V. (2017). Erythrocyte-Platelet Hybrid Membrane Coating for Enhanced Nanoparticle Functionalization. Adv. Mater..

[34] Jiang Q., Liu Y., Guo R., Yao X., Sung S., Pang Z., Yang W. (2019). Erythrocyte-cancer hybrid membrane-camouflaged melanin nanoparticles for enhancing photothermal therapy efficacy in tumors. Biomaterials.

[35] Wang D., Dong H., Li M., Cao Y., Yang F., Zhang K., Dai W., Wang C., Zhang X. (2018). Erythrocyte-Cancer Hybrid Membrane Camouflaged Hollow Copper Sulfide Nanoparticles for Prolonged Circulation Life and Homotypic-Targeting Photothermal/Chemotherapy of Melanoma. ACS Nano.

[36] Dapkute D., Pleckaitis M., Bulotiene D., Daunoravicius D., Rotomskis R., Karabanovas V. (2021). Hitchhiking Nanoparticles: Mesenchymal Stem Cell-Mediated Delivery of Theranostic Nanoparticles. ACS Appl. Mater. Interfaces.

[37] Gao W., Hu C.-M.J., Fang R.H., Luk B.T., Su J., Zhang L. (2013). Surface Functionalization of Gold Nanoparticles with Red Blood Cell Membranes. Adv. Mater..

[38] Guo Y., Wang Z., Shi X., Shen M. (2022). Engineered cancer cell membranes: An emerging agent for efficient cancer theranostics. Exploration.

[39] Liu W.L., Zou M.Z., Liu T., Zeng J.Y., Li X., Yu W.Y., Li C.X., Ye J.J., Song W., Feng J. (2019). Expandable Immunotherapeutic Nanoplatforms Engineered from Cytomembranes of Hybrid Cells Derived from Cancer and Dendritic Cells. Adv. Mater..

[40] Chen H.Y., Deng J., Wang Y., Wu C.Q., Li X., Dai H.W. (2020). Hybrid cell membrane-coated nanoparticles: A multifunctional biomimetic platform for cancer diagnosis and therapy. Acta Biomater..

[41] Zhou H., Fan Z., Lemons P.K., Cheng H. (2016). A Facile Approach to Functionalize Cell Membrane-Coated Nanoparticles. Theranostics.

[42] Muzykantov V.R. (2010). Drug delivery by red blood cells: Vascular carriers designed by mother nature. Expert Opin. Drug Deliv..

[43] Feng S., Li H., Ren Y., Zhi C., Huang Y., Chen F., Zhang H. (2020). RBC membrane camouflaged boron nitride nanospheres for enhanced biocompatible performance. Colloids Surf. B Biointerfaces.

[44] Pisoni D.S., Todeschini L., Borges A.C., Petzhold C.L., Rodembusch F.S., Campo L.F. (2014). Symmetrical and asymmetrical cyanine dyes. Synthesis, spectral properties, and BSA association study. J. Org. Chem..

[45] Sun X., Zhang J., Wang Z., Liu B., Zhu S., Zhu L., Peng B. (2019). Licorice isoliquiritigenin-encapsulated mesoporous silica nanoparticles for osteoclast inhibition and bone loss prevention. Theranostics.

[46] Cao M., Zhan M., Wang Z., Wang Z., Li X., Miao M. (2020). Development of an Orally Bioavailable Isoliquiritigenin Self-Nanoemulsifying Drug Delivery System to Effectively Treat Ovalbumin-Induced Asthma. Int. J. Nanomed..

[47] Liu B., Wang W., Fan J., Long Y., Xiao F., Daniyal M., Tong C., Xie Q., Jian Y., Li B. (2019). RBC membrane camouflaged prussian blue nanoparticles for gamabutolin loading and combined chemo/photothermal therapy of breast cancer. Biomaterials.

[48] Xiang S., Zeng H., Xia F., Ji Q., Xue J., Ren R., Que F., Zhou B. (2021). The dietary flavonoid isoliquiritigenin induced apoptosis and suppressed metastasis in melanoma cells: An in vitro and in vivo study. Life Sci..

[49] Yuan X., Zhang B., Gan L., Wang Z.H., Yu B.C., Liu L.L., Zheng Q.S., Wang Z.P. (2013). Involvement of the mitochondrion-dependent and the endoplasmic reticulum stress-signaling pathways in isoliquiritigenin-induced apoptosis of HeLa cell. Biomed. Environ. Sci.

[50] Chen X., Zhang B., Yuan X., Yang F., Liu J., Zhao H., Liu L., Wang Y., Wang Z., Zheng Q. (2012). Isoliquiritigenin-induced differentiation in mouse melanoma B16F0 cell line. Oxid. Med. Cell. Longev..

[51] Anerillas C., Herman A., Rossi M., Munk R., Lehrmann E., Martindale J., Cui C., Abdelmohsen K., De S., Gorospe M. (2022). Early SRC activation skews cell fate from apoptosis to senescence. Sci. Adv..

[52] Denton D., Kumar S. (2015). Terminal Deoxynucleotidyl Transferase (TdT)-Mediated dUTP Nick-End Labeling (TUNEL) for Detection of Apoptotic Cells in Drosophila. Cold Spring Harb. Protoc..

[53] Moore C.L., Savenka A.V., Basnakian A.G. (2021). TUNEL Assay: A Powerful Tool for Kidney Injury Evaluation. Int. J. Mol. Sci..

[54] Hsia S.M., Yu C.C., Shih Y.H., Yuanchien Chen M., Wang T.H., Huang Y.T., Shieh T.M. (2016). Isoliquiritigenin as a cause of DNA damage and inhibitor of ataxia-telangiectasia mutated expression leading to G2/M phase arrest and apoptosis in oral squamous cell carcinoma. Head Neck.

[55] Gao F.H., Hu X.H., Li W., Liu H., Zhang Y.J., Guo Z.Y., Xu M.H., Wang S.T., Jiang B., Liu F. (2010). Oridonin induces apoptosis and senescence in colorectal cancer cells by increasing histone hyperacetylation and regulation of p16, p21, p27 and c-myc. BMC Cancer.

[56] Zhou G.S., Song L.J., Yang B. (2013). Isoliquiritigenin inhibits proliferation and induces apoptosis of U87 human glioma cells in vitro. Mol. Med. Rep..

[57] Strasser A., Vaux D.L. (2018). Viewing BCL2 and cell death control from an evolutionary perspective. Cell Death Differ..

[58] Maes M.E., Schlamp C.L., Nickells R.W. (2017). BAX to basics: How the BCL2 gene family controls the death of retinal ganglion cells. Prog. Retin Eye Res..

[59] Li C., Zhou X., Sun C., Liu X., Shi X., Wu S. (2019). Isoliquiritigenin inhibits the proliferation, apoptosis and migration of osteosarcoma cells. Oncol. Rep..

