# Supplementary figures and images for: Hybrid Membrane-Derived Nanoparticles for Isoliquiritin Enhanced Glioma Therapy
Source: Pharmaceuticals (Basel). 2022 Aug 26;15(9):1059. doi: 10.3390/ph15091059 (PMC9506545; doi:10.3390/ph15091059)

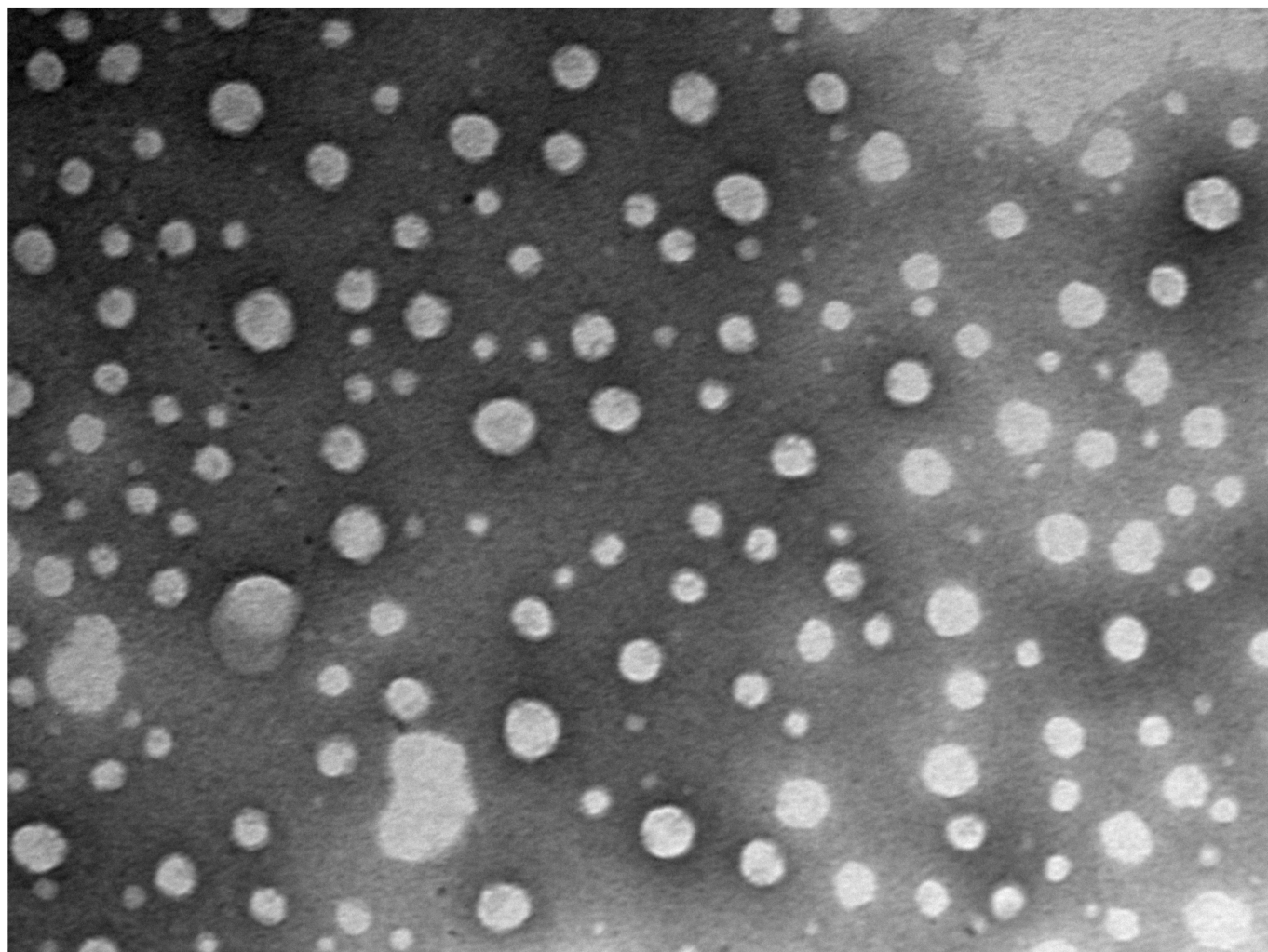

Acc. voltage=100.0kV  
Magnification=x50.0k

200nm

Supplement: Supplementary file 1 [file pharmaceuticals-15-01059-s001.zip › pharmaceuticals-1845517-supplementary.pdf]
